# Compressed Sensing Technique for the Localization of Harmonic Distortions in Electrical Power Systems

**DOI:** 10.3390/s22176434

**Published:** 2022-08-26

**Authors:** Luis Amaya, Esteban Inga

**Affiliations:** 1 Master of Electricity Program, Universidad Politécnica Salesiana, Quito 170525, Ecuador; 2 Master in ICT for Education, Smart Grid Research Group (GIREI), Universidad Politécnica Salesiana, Quito 170525, Ecuador

**Keywords:** compressed sensing, harmonic distortion, dictionary matrix, signal reconstruction, convex optimization, sparse signal

## Abstract

The present work proposes to locate harmonic frequencies that distort the fundamental voltage and current waves in electrical systems using the compressed sensing (CS) technique. With the compressed sensing algorithm, data compression is revolutionized, a few samples are taken randomly, a measurement matrix is formed, and according to a linear transformation, the signal is taken from the time domain to the frequency domain in a compressed form. Then, the inverse linear transformation is used to reconstruct the signal with a few sensed samples of an electrical signal. Therefore, to demonstrate the benefits of CS in the detection of harmonics in the electrical network of this work, power quality analyzer equipment (commercial) is used. It measures the current of a nonlinear load and issues its results of harmonic current distortion (THD-I) on its screen and the number of harmonics detected in the network; this equipment acquires the data based on the Shannon–Nyquist theorem taken as a standard of measurement. At the same time, an electronic prototype senses the current signal of the nonlinear load. The prototype takes data from the current signal of the nonlinear load randomly and incoherently, so it takes fewer samples than the power quality analyzer equipment used as a measurement standard. The data taken by the prototype are entered into the Matlab software via USB, and the CS algorithm run and delivers, as a result, the harmonic distortions of the current signal THD-I and the number of harmonics. The results obtained with the compressed sensing algorithm versus the standard measurement equipment are analyzed, the error is calculated, and the number of samples taken by the standard equipment and the prototype, the machine time, and the maximum sampling frequency are analyzed.

## 1. Introduction

The constant evolution of digital and power electronics means that more and more nonlinear loads are connected to electrical systems. Non-linear loads deteriorate the quality of the energy delivered by electricity distribution companies to their users. Electrical power has become a product and not just a service; therefore, it is quantified by its quality, price, and accessibility characteristics. The quality of the electrical product is measured from quantifiable variables, one of them being the harmonic distortion (THD). This variable measures how much the sinusoidal waveform of voltage or current is deformed concerning its fundamental wave [1,2].

Consequently, to calculate the THD, it is necessary to find the amplitude and frequency of the harmonic waves immersed in the electrical wave, which are frequency multiples of the fundamental signal of the system called harmonics, whose amplitude decreases as the frequency multiplesincreaseand arethe fundamental causesof the distortion of the voltage and current waves [3].

The discrete Fourier transform (DFT) is the classical technique to identify and locate the sine waves of multiple frequencies of the fundamental signal called harmonics [4].

The DFT is based on taking the signal from the time domain to the frequency domain; the frequency domain is when its analysis is performed for harmonics detection. For the DFT to work correctly, it is necessary to sample the signal at least twice the maximum frequency of the harmonic wave according to the Shannon/Nyquist theory.

The present work aims to identify and measure the amplitude and frequencies of the harmonic waves that distort the fundamental electric current signal of a nonlinear load using the compressed sensing (CS) technique. By taking samples less than or equal to the maximum frequency of the harmonic wave, fewer data are taken, and a result similar to that of DFT will be obtained [5,6].

With the compressed sensing algorithm, data compression is revolutionized, a few samples are collected randomly, a measurement matrix is formed, and according to a linear transformation, the signal is taken from the time domain to the frequency domain in a compressed form. The inverse linear transformation is used to reconstruct the signal. An indeterminate system of equations is formed, which is solved through a convex optimization with the L1-norm. As a result of the optimization, a sparse vector is optimized [7].

To demonstrate the benefits of CS in the detection of harmonic waves in the electrical network, a power quality analyzer equipment (commercial), which measures the current of a nonlinear load and delivers the result of total harmonic distortion of the current (THD-I) on its screen, as well as the number of harmonics detected and their amplitude and frequencies, is taken as a measurement standard in this work. Conventional equipment works based on the DFT algorithm and the Shannon–Nyquist theorem [8].

A resistive type of current sensor is connected to the same nonlinear load, which works as a voltage divider; since it is a resistive type of sensor, there are no problems with the bandwidth. It takes data randomly and incoherently through a microcontroller; this way, it takes fewer samples than the power quality analyzer equipment used as a measurement standard [9,10,11,12]. Then, to find even the 32nd harmonic, the classical DFT technique requires a minimum of 64 samples per cycle. With the method proposed in this work using CS, only a number less than or equal to 32 samples per cycle is required to obtain the 32nd harmonic.

Table 1 shows the objective of this research in the localization of harmonics in an electrical network; the CS takes fewer samples per cycle, and the same result is obtained.

**Figure 1:**
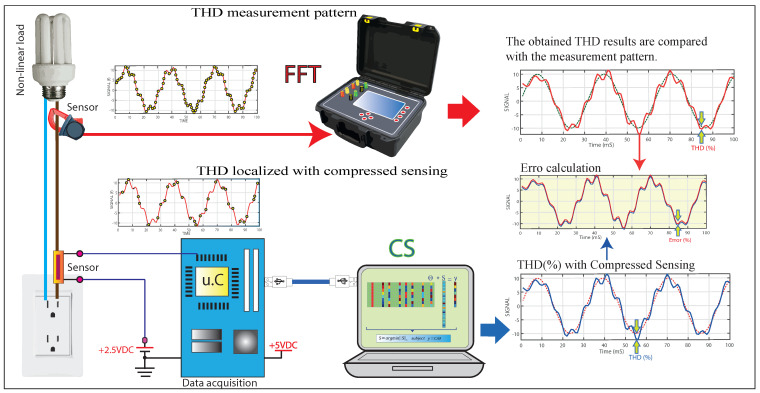
Process diagram of harmonic distortion detection by compressed sensing.

As a result, we have the harmonic distortions of the THD-I current signal and the number of harmonics. The results obtained with the CS algorithm are analyzed versus the measurements obtained with the classical DFT algorithm using the standard measurement equipment. The evaluation parameters of the proposed CS method are the error when comparing the results of CS versus DFT, the number of samples taken by CS and DFT per cycle (sampling frequency), and the machine time taken by each method. The present work is synthesized in Figure 1.

Hereafter, the present work is organized as follows: In Section 2, a review of related works is performed; in Section 3, the problem is formulated and the proposed methodology; in Section 4, the results are analyzed; in Section 5, the conclusions are stated.

## 2. Related Works

In this section, the works related to the present investigation are analyzed, then indicators are extracted from the related results to evaluate the current work. The removed aspects are: the percentage of error obtained when reconstructing the signal under test, the type of measurement matrix, and the linear transformation used. The different applications of compressed sensing are examined, and all the works coincide in calculating sparsity to determine the sparse representation of the signal and to choose the correct linear transformation with which it is possible to compress the electrical signal [13].

The accelerated growth of technology concerning the speed of calculation of computers has revolutionized the paradigm of signal compression; currently, it is not necessary to sense and store large amounts of data; it is currently possible to sense and store few data and have the same information when processing large amounts of data. Thus, to achieve the compression of a signal, the current trend is the use of the compressed sensing technique, which is developed within the mathematical fields of linear optimization and descriptive statistics [1,14].

One of the most relevant characteristics of the compressed sensing technique is data storage in the frequency domain. From the state-of-the-art survey, most of the research works concerning compressed sensing (CS) deal with audio, image, and video applications [15,16].

Furthermore, also, CS is applied to detect harmonics in frequency-dispersive electrical signals from ships; the Bernoulli-type measurement matrix with different probabilistic values is used; the discrete Radon transform (DRT) is used as a linear transform. The use of the DRT requires numerous measurements, which detract from the compressed sensing technique. Consequently, to mitigate this disadvantage, a K-rank filter is used in the domain of the linear transform, thus compressing the signal under analysis with a fast convergence, which implies a low computational time for the calculation [17,18].

The CS is also applied to estimate the state of electrical distribution systems, using data from synchrophasor measurement units (PMUs) installed in an electrical system. This application proposes a novel and simple algorithm called DSSE, which solves a set of linear equations without any iterative process. It uses the IEEE 123-bus power system (SEP), which locates synchrophasor measurement units (PMUs) in some busbars of the SEP with which it measures the voltage in magnitude and angle in the busbars, and the PMU is installed by compressed sensing. It estimates the magnitude and angle voltage values of the other busbars of the system when no synchrophasor measurement units are installed. The indeterminate system of equations is formed between the number of busbars and the number of PMUs installed in the SEP, with the number of busbars being more significant than the small number of PMUs installed in the SEP. The indeterminate system of equations is solved by a nonconvex linear optimization problem using the L1-norm. The Bernoulli-type measurement matrix and the discrete Fourier transform are used. Finally, the signal reconstruction error is 0.5% [19].

A mathematical application of the CS is to perform a critical appraisal of the purely harmonic analysis of the CS compressed sensing theory of Candes, Romberg, and Tao. It is applied to the determination of a trigonometric series called “Lacunar”, whose sum is known over an interval. It demonstrates how purely harmonic analysis methods can obtain preliminary results and how some classical or new problems of harmonic analysis are related to compressive sensing. It uses DFT as a dictionary matrix and solves the under-determined system of equations with the minimization of the L2-norm [20].

The harmonic pollution of electrical networks is becoming increasingly complex, making it necessary to continuously monitor the power quality, encountering a problem with the amount of data to be stored and processed. A solution to the data storage problem is through compressed sensing. It is possible to simultaneously perform the functions of compressive sampling, signal reconstruction, and harmonic detection functions. The dispersion of the harmonic signals is calculated numerically; the Gaussian matrix is used as a measurement matrix; the discrete Fourier transform is used as the basis for the linear transformation. The indeterminate system of equations is solved by a unique method called the spectral projected gradient with fundamental filter (SPG-FF). Finally, a signal reconstruction error of 1.8% is obtained [21].

Another related work tests the integral nonlinearity (INL) and randomly measures the output voltages by forming a subset of the data from the digital-to-analog converters (DACs). Compressed sensing recovers the INL values of all input codes using a Gaussian measurement matrix and the discrete cosine transform as a linear transform. This method is implemented practically and evaluated experimentally with two DACs of different architectures; the recovery of the curve is very accurate with respect to the INL curve obtained by a standard method and used as a measurement standard in this way to perform the metrics of comparison of the model [22,23].

A related application to the present work uses a wireless sensor network to monitor freshwater quality in a container. It does not use the usual measurement nuances; instead, it uses continuous models for both the signal acquisition process and the sampling process, arbitrarily taking the data; it does not need a trained dictionary and, therefore, does not use a signal database. It makes the algorithm more accurate, robust, and stable in the presence of white noise and reduces the power consumption of the sensors that monitor water by 25%. An update of the firmware is used in this model with a 12 bit resolution of the sensors. Thus, the authors claim that they could reduce the power consumption of the sensors by 60% [24,25].

Table 2 shows a comparative summary of the related works versus the present case study, taking as indicators: the percentage of error obtained when reconstructing the signal under test, the type of measurement matrix, the practical and theoretical application, and the linear transform used.

## 3. Problem Formulation

**Figure 2:**
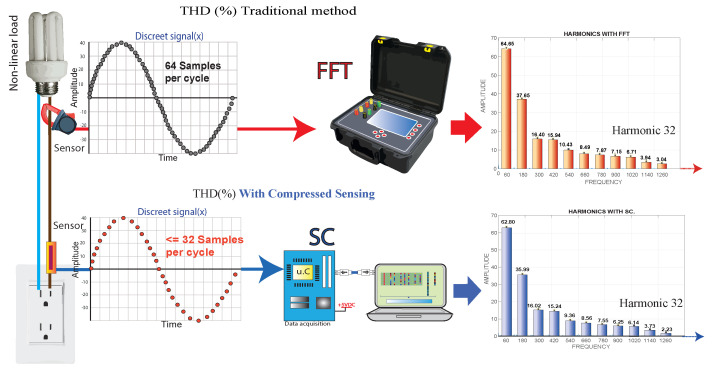
Methodology used for the problem formulation.

The objective of this article is the detection of harmonic frequencies of the current signal of a nonlinear load; with the harmonic frequencies obtained, the total harmonic distortion (THD) is calculated, and all this methodology is executed through compressed sensing (CS). The nonlinear load under study is measured (THD) with conventional measuring equipment (standard measurement) and the prototype object of this work. It is shown that by using CS in the localization of harmonic frequencies, the number of samples per cycle is reduced by less than 50%, reducing memory consumption and the data processing load, achieving a high speed of analysis and processing of the proposed algorithm. The conventional equipment bases its operating principle on the discrete Fourier transform (DFT) and the Shannon/Nyquist theory, which state that to reconstruct a signal, it is necessary to take samples of the signal at least two-times the maximum frequency of the fundamental wave. The proposed prototype bases its operation on the compressed sensing technique, which uses a measurement vector, a dictionary matrix, and a convex optimization method; a nonlinear load current signal is reconstructed from randomly sampled incoherent linear measurements involving the acquisition of broadband data below the Shannon–Nyquist sampling frequency. For the proposed study, conventional equipment, to obtain up to the 32nd harmonic frequency, needs to take at least 64 samples per cycle. The proposed CS-based prototype, to obtain the 32nd harmonic frequency, will take anumber equal to 32 samples per cycle. The conventional equipment and the proposed prototype can obtain the harmonic frequency number 32. For the present case study, the maximum harmonic frequency obtained is 21, as illustrated in Figure 2.

### 3.1. Proposed Strategy and Methodology

The mathematical methodology that uses compressed sensing is not complex to state, but it is complex to solve since it forms a problem of type NP. Thanks to the accelerated growth in computer technology that we have today, it has been possible to solve with relative speed the indeterminate systems of equations formed using the compressed sensing technique [7].

**Figure 3:**
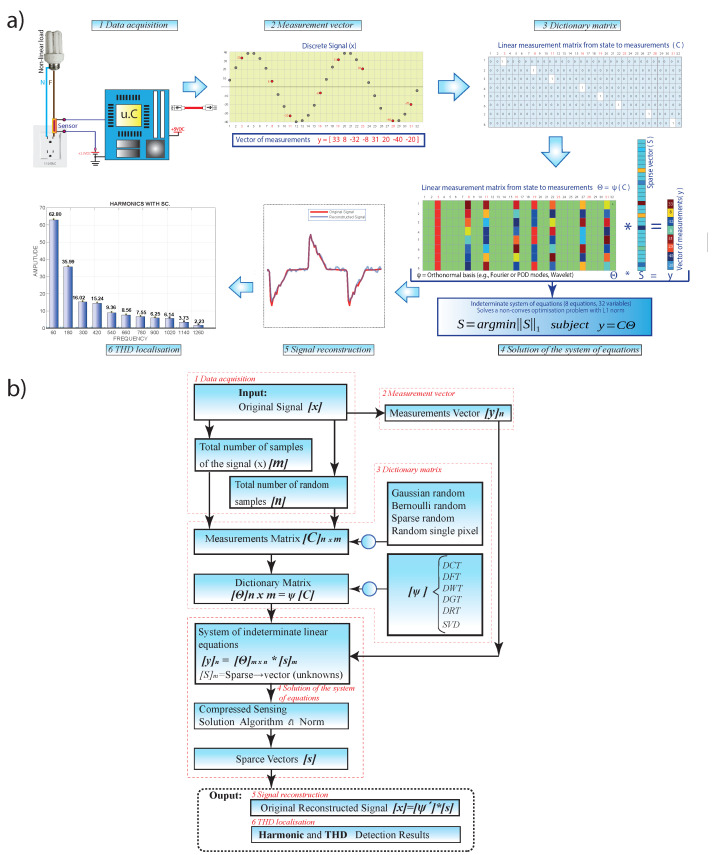
(**a**) General block diagram and (**b**) flow diagram of the proposed model.

The methodology used in this work is explained step by step and is divided into six parts: data acquisition, measurement vector, dictionary matrix, solution of the system of equations, signal reconstruction, and THD localization. Figure 3 shows a general diagram of the proposed methodology for detecting harmonic frequencies with compressed sensing.

#### 3.1.1. Data Acquisition

The data to be treated were acquired using a resistive-type current sensor. The linearization of the voltage drop existing in the sensor was carried out so that it was directly proportional to the current consumed by the load. This means that the time variation between the acquisition of one datum concerning another was different, 20, and the magnetic field present in the environment inside in the operation of these sensors, causing the reference to be displaced; for this reason, Hall effect sensors were not used. From practical experimentation, it was determined that the Hall effect sensors did not have a sufficient wavelength to determine harmonic frequencies higher than harmonics of 20. The magnetic field in the insulated environment was insufficient to decide on the harmonic frequencies. Therefore, using the resistive sensor, the voltage drop signal was acquired. It is an analog-type signal whose values vary at the frequency of the network, taking positive values during half the cycle and negative values in the following half of the cycle, in a range of plus or minus 1 volt. The microcontroller used does not have any capacity to work with analog signals with negative cycles, so it is necessary to couple the analog signal. This coupling was performed by adding 2.5 volts in direct current to the sensed signal; in this way, the signal reference was 2.5 volts continuous, and the value of the negative half cycle now ranged from 1.5 VDC to 2.5 VDC; the ADC analog–digital converter of the microcontroller has a resolution of 12 bits; this digitized signal is sent via USB communication to the algorithm implemented in Matlab.

#### 3.1.2. Measurement Vector

The discrete signal (x) acquired by the microcontroller at random was input to the compressed sensing algorithm [28].

**Figure 4:**
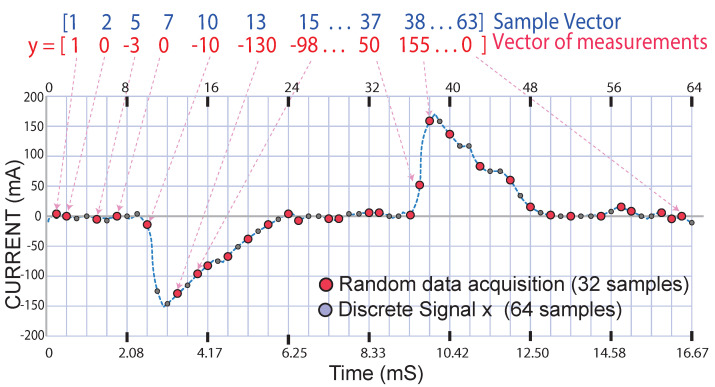
Obtaining the measurement vector of the discrete signal (x) in the time domain (t).

The discrete signal (x) is a vector having 64 samples; from this vector, 32 samples were extracted randomly and incoherently, and with the 32 acquired samples, a vector of measurements (y) was formed, as illustrated in Figure 4.

From a mathematical point of view, compressed sensing exploits the signal’s sparsity on a generic basis Ψ, thus achieving complete signal reconstruction from a reduced number of measurements [29].

If a signal (x) is K sparse in (y), instead of measuring the signal (x) directly with (n) measurements and then compressing, it is possible to collect a smaller number of randomly chosen sizes and then resolve the nonzero elements of (s) in the transformed coordinate system [20].

The (y) measurements ∈ Rp, with K < p, and n are given by Equation (1):

(1) $$\begin{array}{c} {\left[y\right]}_{1*n}={\left[C\right]}_{m*n}\times {\left[x\right]}_{1*m} \end{array}$$

where ${\left[y\right]}_{1*n}$ is the vector of measurements. ${\left[x\right]}_{1*m}$ is the discrete signal under test. ${\left[C\right]}_{m*n}$ is the measurement matrix. For this case study, $\left[m=64\right]$ and $\left[n=32\right]$

#### 3.1.3. Matrix Dictionary

Within compressed sensing, there is a matrix called a dictionary ${\left[\Theta \right]}_{m*n}$, which is formed by a measurement matrix ${\left[C\right]}_{m*n}$ and a linear transformation, also called a generic base (Ψ). Depending on the desired application of compressed sensing, the measurement matrix and the linear transformation are chosen.

The success of compressed sensing is to choose the measurement matrix ${\left[C\right]}_{m*n}$ that is sufficiently incoherent concerning the transformation basis (Ψ) [30].

The most-common measurement matrices are the random single pixel, Gaussian random, Bernoulli random, and Sparse random matrices. The use of each of these matrices depends on the intended application. For the present work, the random single-pixel measurement matrix was used; with this matrix, the coefficients were classified, extracting the most-representative ones and converting the less-representative ones to zero; in this way, it is possible to compress high-dimensional vectors in a low-dimensional space, preserving the spectral properties. Using Gaussian random, Bernoulli random, and Sparse random matrices did not achieve data compression [31].

To form the single-pixel random-type medicines matrix (c), it takes the total number of data acquired in the measurement vector (y)(n = 32), 32 rows, and the total number of data contained in the discrete signal (x) (m = 64), 64 columns, so the matrix C is composed of 32 rows and 64 columns.

Each row of the matrix represents a vector containing 64 elements, of which 63 elements = 0 and one element = 1. The ordered pair to locate the number 1 in the matrix is obtained as follows.

Row 1 takes the first element of the sample vector, number 1; then, in column 1, row 1, the number 1 is placed.

Row 2 takes the second element of the sample vector, number 2, then in column 2, row 2, the number 1 is placed.

Row 3 takes the sample vector’s third element, the number 5, then in column 3, row 5, the number 1 is placed, and so on.

**Figure 5:**
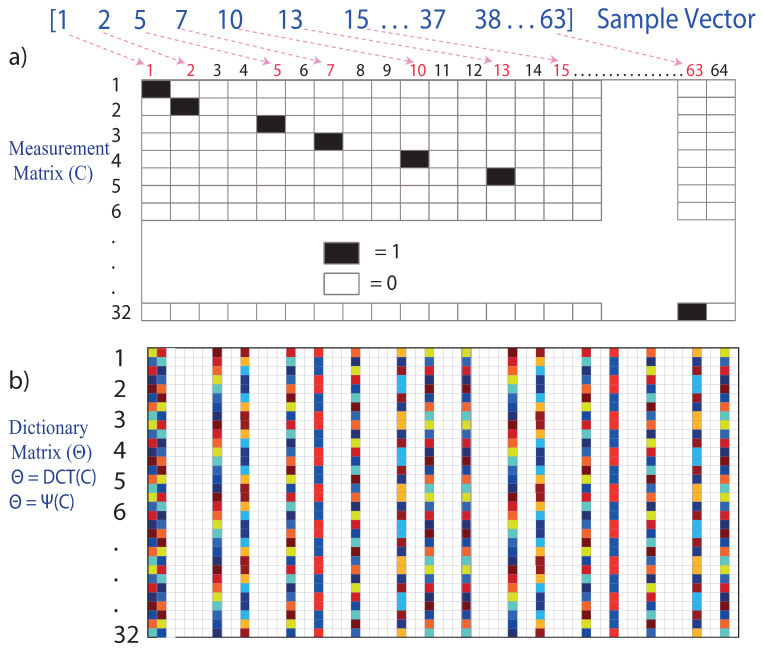
(**a**) Measurement matrix (C). (**b**) Dictionary matrix (Θ).

Row 32 takes the last element of the sample vector, which is the number 63; then, in column 32, row 63, the number 1 is placed. This is illustrated in Figure 5a.

**Figure 6:**
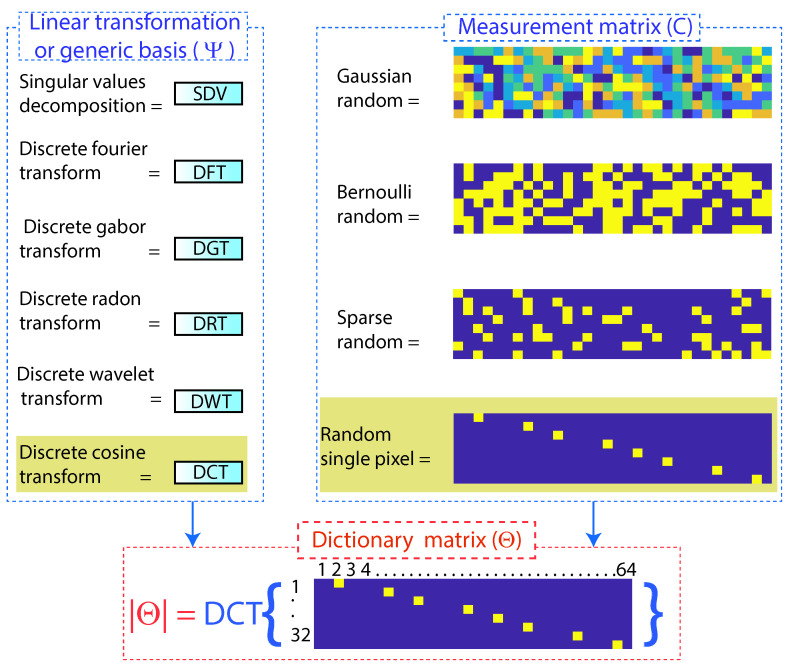
Formation of the dictionary matrix (Θ).

The measurement matrix C∈Rp∗n represents a set of (p) linear measurements on the state (x). The measurement matrix ${\left[C\right]}_{m*n}$ choice is important in compressed sensing, as illustrated in Figure 6. Typically, the measurements may consist of random projections of the state, in which case the entries of ${\left[C\right]}_{m*n}$ are random variables with a Gaussian or Bernoulli distribution [32].

A generic basis, such as Fourier or wavelets coupled with the random single-pixel single point measurement matrix, is ideal for signal compression since these are incoherent concerning these bases, obtaining a broadband frequency response.

Once the measurementmatrix ${\left[C\right]}_{m*n}$ is obtained, the dictionary matrix ${\left[\Theta \right]}_{m*n}$ is created by means of a linear transformation; for the present case study, the discrete cosine transform (DFT) was used, where a sufficient number of good measurements results in a dictionary matrix ${\left[\Theta \right]}_{m*n}$, as illustrated in Figure 5b and according to Equation (2), which preserves the distance and the structure of the inner product of the sparse vectors ${\left[s\right]}_{m}$ [19].

For the work under study, the discrete cosine transform (DCT) was used as the basis of transformation Ψ, mathematically transforming the single-pixel random measurementmatrix ${\left[C\right]}_{m*n}$ from the time domain to the frequency domain according to Equation (2).

(2) $$\begin{array}{c} {\left[\Theta \right]}_{m*n}=DCT\left\{{\left[C\right]}_{m*n}\right\}\to DCT=\Psi \end{array}$$

#### 3.1.4. Solution to the Indeterminate System of Equations

Having obtained the dictionary matrix ${\left[\Theta \right]}_{m*n}$, the vector of measurements ${\left[y\right]}_{1*n}$ forms a system of linear equations when the unknown is the sparse vector ${\left[s\right]}_{1*m}$. Therefore, compressed sensing aims to find the most-sparse vector ${\left[s\right]}_{1*m}$ consistent with the measurements ${\left[y\right]}_{1*n}$. A measurement matrix ${\left[C\right]}_{m*n}$ is used as a near-isometry map over the sparse vectors.

Isometry means the same distance closely related to unitarity, preserving the length and angles between vectors. When acting as a close (same distance) isometry, it is possible to solve Equation (3) to find the most-sparse vector ${\left[S\right]}_{1*m}$ using L1 convex minimization since the system of equations is under-determined, with infinitely many consistent solutions for ${\left[S\right]}_{1*m}$ [33].

(3) $$\begin{array}{c} {\left[y\right]}_{1*n}={\left[C\right]}_{m*n}*\left[\Psi \right]*{\left[s\right]}_{1*m}={\left[\Theta \right]}_{m*n}*{\left[s\right]}_{1*m} \end{array}$$

**Figure 7:**
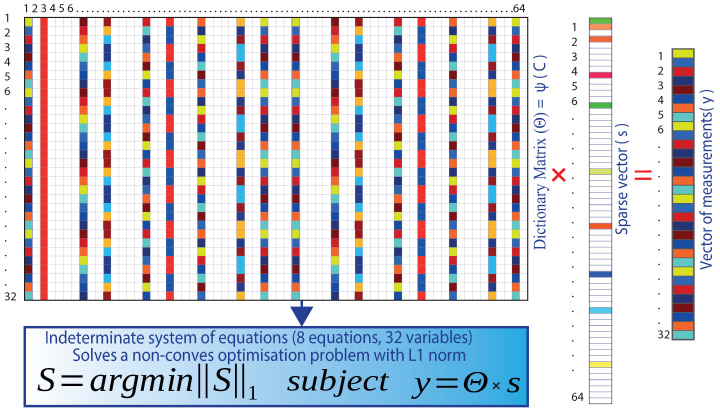
Indeterminate system of equations and its solution with the L1-norm.

For the case of the study, the dictionary matrix ${\left[\Theta \right]}_{m*n}$ is made up of 32 rows and 64 columns; there are 32 equations and 64 variables; the measurement vector ${\left[y\right]}_{1*n}$ has 32 rows and one column. The unknown is the sparse vector ${\left[s\right]}_{1*m}$ of 64 rows and one column. The reason for compressed sensing in this work is that the sparse vector ${\left[s\right]}_{1*m}$ has as many zeros as possible, hence the name “sparse”. Figure 7 shows the indeterminate system of equations of the example case.

It becomes a linear optimization problem (convex minimization of L1), where Equation (4) finds the sparsest solution for (s) that satisfies the optimization problem [33]:

(4) $$\begin{array}{c} \hat{s}=\underset{s}{argmin{∥S∥}_{1}}\;subject\to {\left[y\right]}_{1*n}*\Psi *{\left[s\right]}_{1*m} \end{array}$$

where ${∥.∥}_{1}$ is the L1-norm given by Equation (5):

(5) $$\begin{array}{c} {∥S∥}_{1}=\sum_{k=1}^{n}\left|Sk\right| \end{array}$$

**Figure 8:**
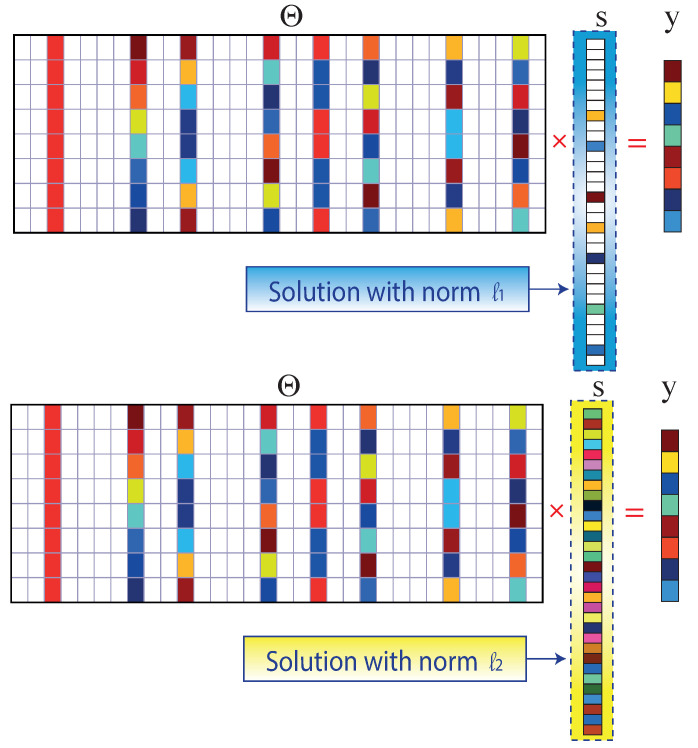
L1 and L2 minimum standard solutions to the compressed sensing problem.

The minimum L1-norm solution is sparse, while the minimum L2-norm solution is sparse [33]. The linear indeterminate system of equations (Equation (4)) can be solved using the L2-norm with particular conditions that must be met for the L2 minimization to converge, but the solution is not sparse, as illustrated in Figure 8.

If white noise is immersed in the signal under test, Equation (5) should be varied to obtain the more robust Equation (6). With more samples per cycle, the system gains robustness to noise. Furthermore, the system is more robust to noise if a sufficiently large dispersity level k is used, i.e., a more significant number of cycles to be analyzed. Thus, Equation (6) is included in the algorithm to mitigate the noise immersed in the signal. The error also depends on the dictionary used; therefore, when a random type of measurement matrix is used, as is the case in this work, and a random single pixel is used as a measurement matrix, it is recommended to perform a noise analysis since it influences the error at the time of signal reconstruction. Thus, the total harmonic distortion (THD) error [34]:

(6) $$\begin{array}{c} \hat{s}=\underset{s}{argmin{∥S∥}_{1}}\;subject\to {∥\Psi *{\left[s\right]}_{{}_{1*m}}-{\left[y\right]}_{1*n}∥}_{2}<\varepsilon \end{array}$$

#### 3.1.5. Signal Reconstruction

Once found, the sparse vector (s) that contains the signal (x) is in compressed form in the frequency domain; when passing the vector (s) to the time domain with Equation (7), it is possible to reconstruct the original signal with an excellent approximation [35].

The approximation error is analyzed in the following section, with an actual signal measured by a data acquisition card; the acquired data are processed with the compressed sensing algorithms proposed in this work and compared with the additions obtained by a commercial power quality instrument, which will serve as a measurement standard [36].

(7) $$\begin{array}{c} Signal\left(x\right)={\left[x\right]}_{1*m}\approx \Psi^{\prime }\left\{{\left[s\right]}_{1*m}\right\}\approx IDCT\left\{{\left[s\right]}_{1*m}\right\} \end{array}$$

The algorithm used to give a solution to the indeterminate system of Equations (3) is the one that imposes the magnitude of error when reconstructing the signal. According to the state-of-the-art analyzed, the algorithm that presents minor errors when reconstructing the signal in CS is basis pursuit (BP); it is based on the L1-norm, which works by convex optimization and provides a vector (S) that it sufficiently scarce with the majority of its elements equal to zero; in this way, the signal is compressed [31]. Therefore, the basis pursuit (BP) algorithm was used in the present work to solve the indeterminate system of equations and, thus, obtain the least error when reconstructing the signal.

Other algorithms also give solutions to the indeterminate system of Equation (3); these algorithms are used for other applications such as the detection, classification, or estimation of parameters, in which a complete reconstruction of the signal, the most used, does not need to be performed.

Least squares (LQ): This algorithm is based on the least-squares method, searches for a function f(x) that is a linear combination of the basis function, uses the L2 minimization rule, and obtains a sparse vector (S) affecting the signal compression [17].

Orthogonal matching pursuit (OMP): This is based on successive approximations of the signal coefficients by iteratively debugging a sparse solution until convergence is reached. This algorithm is ideal for reconstructing signals with high white noise; its drawback is the increased use of computational resources [37].

Greedy algorithms: The convex optimization method is the most suitable for reconstructing signals by its sparse representation; there are also the so-called iterative greedy algorithms, which have several qualities that, depending on the application, can improve the performance of convex algorithms; these algorithms are based on iterative approximations of the coefficients of the signal, to meet specific convergence criteria. The greedy algorithms are divided into two types: greedy pursuit and thresholding algorithms [36].

Compressive sampling matching pursuit (CoSaMP): This algorithm is also used for signal reconstruction; its margin of error is infinitely more significant than the convex algorithm. CoSaMP was developed by Needell and Tropp [27]. The algorithm initializes with a trivial approximation at each iteration; then, it calculates the dot product of the residual with the dictionary matrix (Θ). Additionally, it identifies the most-significant components, thus solving the least-squares problem to approximate the signal with the bound support. It retains only the most essential elements of the least-squares approximation. The samples are updated at each iteration, and their residual is calculated [38].

#### 3.1.6. THD Harmonic Distortion Location

Harmonic distortion frequencies were extracted from the reconstructed current signal through compressed sensing using the discrete Fourier transform (DFT). Fourier analysis of a periodic function removes a series of sines and cosines called harmonics.

Consequently, to measure the total harmonic distortion of the current, the physical quantity called the total harmonic distortion (THD I) was used, proportional to all harmonic components present in a waveform. The admissible value in a power quality analysis is that the THD I be less than or equal to 5%; if this value is exceeded, the signal is contaminated with harmonic frequencies [21]. For the example case, Equation (8) defines the total harmonic distortion in the current waveform of an energy-saving lamp.

(8) $$\begin{array}{c} THD_{I}=\frac{\sqrt{\sum_{h=2}^{h=max}{\left(I_{h}\right)}^{2}}}{I_{1}} \end{array}$$

An error is made whenever a measurement or estimation of a quantity is produced. Once the harmonic frequencies and the percentage of total harmonic distortion of the current waveform have been obtained, the error is calculated according to the parameters obtained and compared with the standard measurements available. Two types of errors are distinguished: The first is the absolute error, ε, Equation (9), which is defined as the positive difference between the typical measurement value and the value reconstructed using the compressed sensing model [15].

(9) $$\begin{array}{c} \varepsilon =\left|I-I_{approx}\right| \end{array}$$

The relative error is defined as the quotient of the absolute error and the common measurement value, as shown in Equation (10).

(10) $$\begin{array}{c} \eta =\frac{\left|I-I_{approx}\right|}{I} \end{array}$$

The relative error can be expressed as a percentage by Equation (11), which can be defined as a percentage.

(11) $$\begin{array}{c} \delta =\eta *100\% \end{array}$$

Calculating the error is the way to determine the model’s adequate parameters, the number of samples per cycle, and the number of samples to be reconstructed in the number of cycles to be considered.

The table of variables used in the mathematical model and the pseudocode of Algorithm 1 are presented in Table 3.
**Algorithm 1** Harmonic detection with the CS algorithm.**Step:1,**Signal acquisition (x):**Step:2.**Definition of parameter:NMP → Number of random samples per periodNP → Number of periods to analyzem → Total number of samples of the signal (x)n → Total number of random samples**Step:3.**[y] → Get measurement vector.[t] → Get time vector.**Step:4.**Formation of the measurement matrix [C].**Step:5.**Dictionary matrix formation:${\left|\Theta \right|}_{m*n}=\Psi \left|C\right|=DCT\left(C\right)$Ψ=discretecosinetransform(DCT)**Step:6.**Formation of the system of indeterminate linear equations with n equations and m variables; m > n.${\left[y\right]}_{n}={\left|\Theta \right|}_{m*n}*{\left[s\right]}_{m}$$Were:{\left[s\right]}_{m}=Sparse\to vector\left(unknowns\right).$**Step:7.**Solution of the indeterminate system of equations by the L1-norm using the L1 magic library.**Step:8.**Reconstruction of the signal:${\left[x^{\prime }\right]}_{m}=\Psi^{\prime }{\left[s\right]}_{m}=IDCT{\left[s\right]}_{m}$$Where:{\left[x^{\prime }\right]}_{m}=Reconstructed\to signal\left(x\right)$**Step:9.**Extraction of harmonics from the reconstructed signal:$No.harmonics=DFT{\left[x^{\prime }\right]}_{m}$**Step:10.**Calculation of harmonic distortion (THD)Calculation of error percentage (%ε).

## 4. Analysis of Results

Therefore, to test the mathematical model developed, an actual current sample was taken from an energy-saving light bulb with commercial power quality analyzer equipment; the measurements acquired with this equipment serve as a measurement standard for the analysis to be performed in this section, and the measurements obtained are shown in Table 4.

Three fundamental variables determine the error of the total harmonic distortion of the current (THD I); these variables are the number of samples taken for each cycle, the number of cycles to be analyzed, and the number of samples of the reconstructed signal (m).

**Figure 9:**
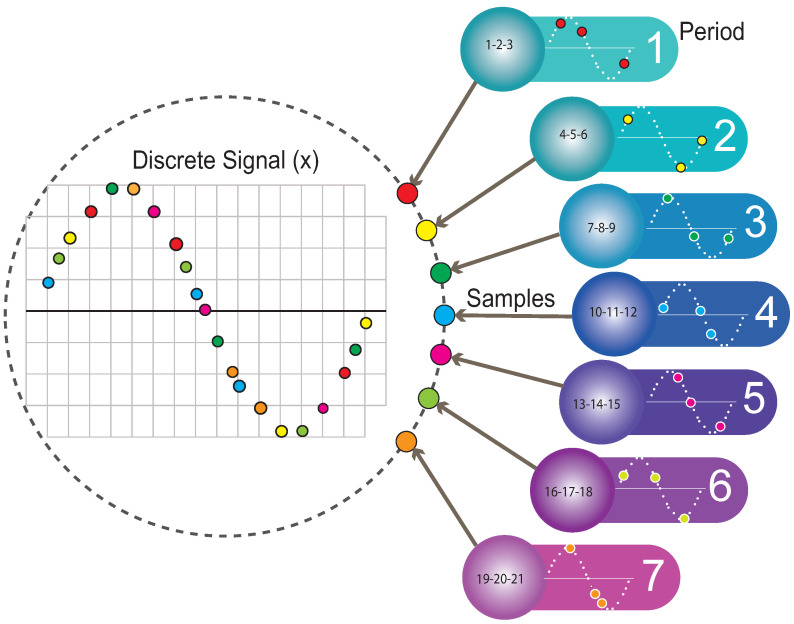
Signal reconstruction parameters and variables.

The model’s effectiveness depends on the number of cycles analyzed and how many random samples are taken in each cycle. Figure 9 illustrates how the signal is formed by merging three random samples per cycle in seven processes. Ideally, the random samples of each cycle should not coincide; in this way, the error of the reconstructed signal is low, but if the pieces overlap between processes, the error of the reconstructed signal rises.

The model analysis starts with the number of samples per cycle constant in 32 samples, and the number of cycles to be analyzed varies from one process to 20 processes. Therefore, the number of samples of the signal to reconstruct is the multiplication of the number of samples to rebuild by the number of cycles; it will start in 64 samples and will culminate in 1280 samples at 20 cycles; this implies that the dictionary matrix begins with 32 rows and 64 columns and ends in 32 rows 1240 columns, while as the analysis cycles increase, the computer calculation time increases.

**Figure 10:**
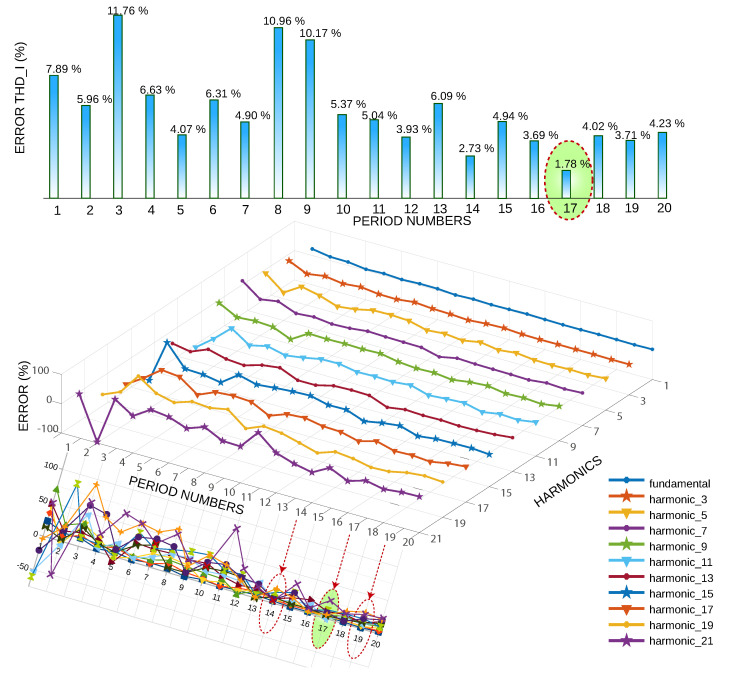
THD error vs. several cycles and harmonic error vs. several cycles.

This depends on how the model took the random samples. For each cycle, the total harmonic distortion of the current (THD I) is calculated, then the relative error is calculated as a function of the standard value of THD I. The higher the harmonic frequency, the greater the error fluctuation; harmonic 21 is the highest error, while the fundamental frequency has the lowest error. Figure 10 shows the 20 runs of the model, obtaining the highest error at three cycles and the lowest error at 17 cycles. An error of less than 4% is achieved in cycles 14, 17, and 19.

**Figure 11:**
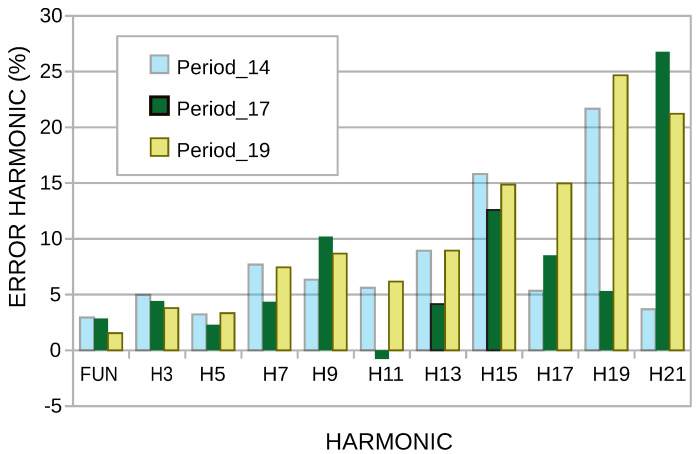
Harmonic error rate of periods 14, 17, and 19.

Figure 11 analyzes the relative error of the harmonics in the periods 14, 17, and 19, the minor error being in the fundamental frequency, and the harmonics of greater frequency are those that have a more significant error, analyzed for 17 periods; it is observed that the more substantial error is in the harmonic 21 and the minor error in harmonic 11.

**Figure 12:**
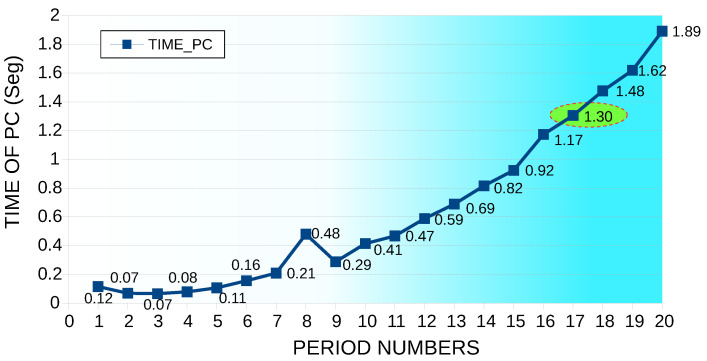
Machine time for a constant number of samples at 32 and varying the number of periods from 1 to 20.

It is essential to analyze the machine time; the model was run under the Matlab software in a PC Intel® Core (TM) I7-6500 CPU-2.50 GHz RAM of 8.00 GB to simulate the number of samples constant in 32 and varying the number of periods from 1 to 20. The machine times are illustrated in Figure 12; since the least error was found at 17 periods, this time was taken as the reference (1.304879 s).

**Figure 13:**
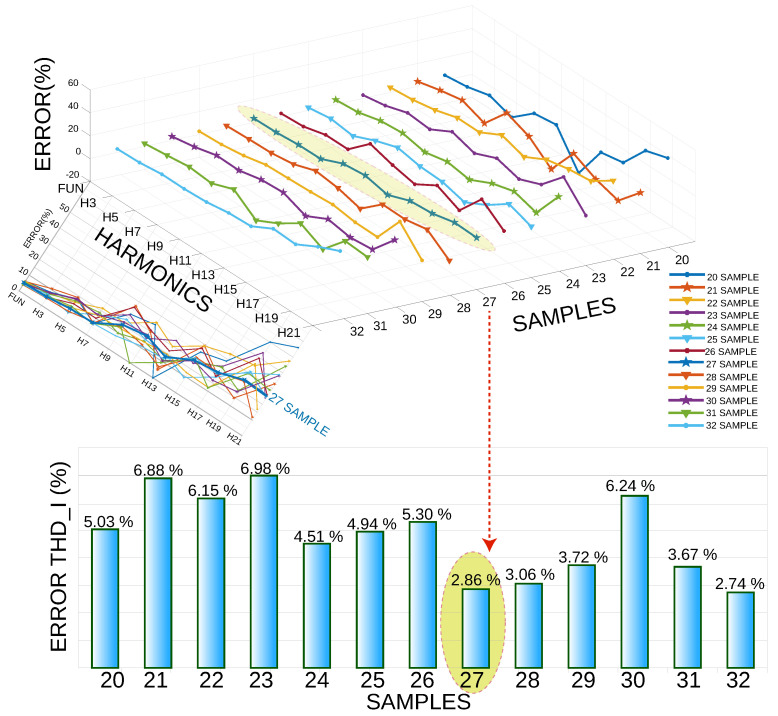
Error vs. harmonics, keeping the number of periods constant at 20 and varying the number of samples per cycle from 20 to 32.

Consequently, a second analysis was performed in which the number of periods was kept constant at 20. Furthermore, the number of samples per cycle varied from 20 representatives to 32 pieces, as illustrated in Figure 13. It was observed that with 27 samples per cycle, a relative THD I error of 2.86% was obtained, and the highest relative error was obtained with 23 pieces per cycle.

**Figure 14:**
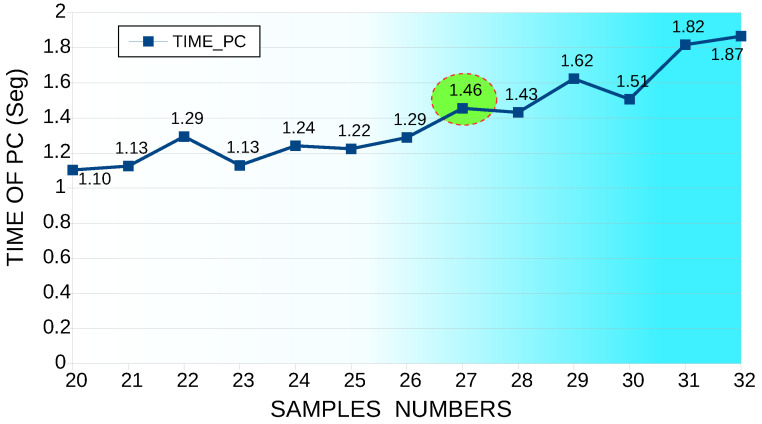
Machine time, number of periods constant at 20, varying the number of samples per cycle from 20 to 32 samples.

Regarding the computational time in this simulation, the number of periods remained constant at 20; additionally, the number of samples varied per cycle from 20 to 32 (Figure 14). According to the simulation process, the lowest error was obtained with 27 samples, for which a machine time of 1.4553 s was used.

From the two simulation cases, the THD I error percentage of the first case was 1.78%, while the THD I error rate of the second case was 2.86%. In the same way, the computational time of THD I of the first case was 1.304879 s. Moreover, the second case was 1.4553 s. Therefore, the first case was the most efficient because it had a minor error and less computational time.

**Figure 15:**
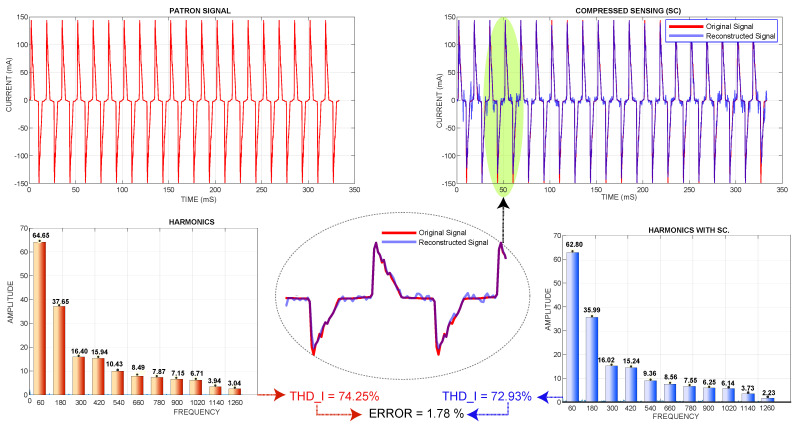
Real signal and its harmonics vs. CS-reconstructed signal and its harmonics.

Figure 15 shows the actual current standard signal and its harmonics versus the signal reconstructed by compressed sensing; its harmonics and the relative error also showed the reconstruction of the signal; this graph corresponds to 17 cycles and 32 samples per cycle.

**Figure 16:**
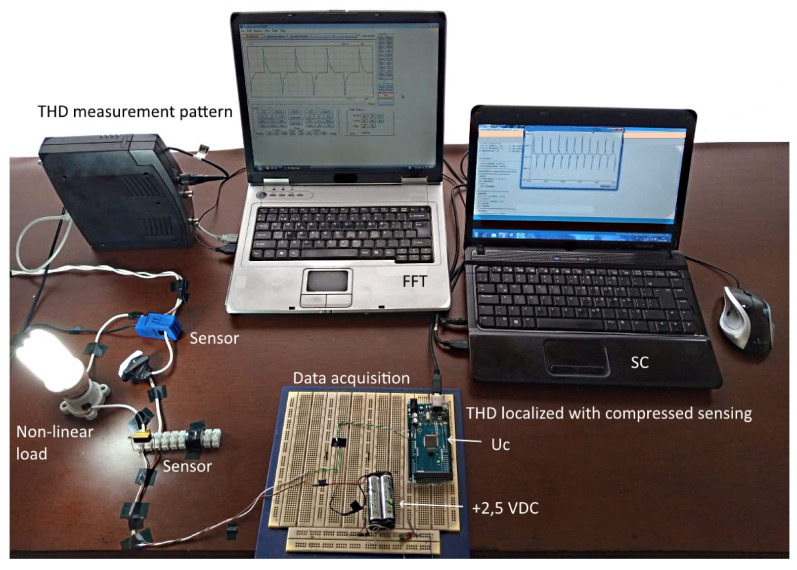
Hardware implemented in the experimental process.

This article contributes to the localization of current harmonic distortion in a nonlinear load, the hardware. It was analyzed how the signal reconstruction error affects the THD error found by compressed sensing compared to the TDH measured by the standard measurement equipment. Figure 16 was physically implemented, and the measurements obtained were compared with the measurements of the standard measurement equipment. Several simulations were performed, varying the number of samples per cycle. In the state-of-the-art, the error rate is mentioned; however, it is not how many processes are being analyzed or how many pieces per cycle are being taken; how it affects the error rate is not included.

The main advantage of the algorithm proposed in the present work is that it allows the sampling of sparse signals with few data, and the signal can be reconstructed in detail, with a high response speed of the algorithm and a low consumption of computational resources, especially in data storage. Therefore, the power quality is comprehensively monitored by collecting a large amount of data in a small storage space.

## 5. Conclusions and Discussion

A signal was taken from the time domain to the frequency domain through a linear transform basis; in this domain, the signal was analyzed for how sparse it was. The compressed sensing technique can reconstruct the sparse signal using a few random measurements.

For the case study proposed in this work, the lowest relative error obtained from the total harmonic distortion of the current THD I of a saver lamp was 1.78%, taking 32 samples per cycle and analyzing 17 processes. Consequently, a system of indeterminate linear equations of 32 equations and 544 variables was formed and solved using the L1 magic library with a machine time of 1.304879 s.

Thus, reconstructing a signal and obtaining the total harmonic distortion through the compressed sensing theory were performed by taking several samples equal to their maximum frequency, in contrast to the Shannon/Nyquist theory. In reconstructing a signal, it is necessary to sample the signal at least two-times the maximum frequency of the fundamental wave.

The efficiency of compressed sensing to locate harmonic distortions in an electrical signal depends on correctly choosing a dictionary matrix composed of a measurement matrix and a linear transformation. After experimenting with several measurement matrices and linear transforms, it was concluded that the lowest error was achieved using the random single-pixel measurement matrix and the discrete cosine transform DCT as the linear transform.

In running the compressed sensing algorithm developed for the present work, its result was continuously varied; this is because the data were taken randomly, fluctuating with a margin of error of +/−2%.

The work presented was migrated to an embedded computer system, and it is planned to use an ODROID-U3 card; this card is based on ARM, capable of running Linux applications, in which Python and all its free libraries are installed, in such a way that we will have a power quality analyzer equipment based on compressed sensing and entirely with free software and hardware.

Future work can start by creating a non-random algorithm for data acquisition that does not conflict with the theory of compressed sensing. This will reduce the fluctuation of the reconstruction error of a periodic signal and reduce the sampling and analysis periods, thus avoiding dependence on access to total high-dimensional measurements, thus reducing the computational time [31,37].

## Figures and Tables

**Table 1:** Differences in harmonic detection between DFT and CS.

	Samples per Cycle	Visualize the Harmonic No.
The classical method for harmonic detection using DFT	Greater than 64	32
The proposed method for harmonic detection by compressed sensing (CS)	Less than 32	32

**Table 2:** Summary of related works and the novelty of this work.

		Measurement Matrix			Linear Transformation			Application		Results
**Author, Year**	**Objectives**	**Bernoulli**	**Gaussian**	**Single Pixel**	**DFT**	**DCT**	**DGT**	**Theoretical**	**Practical**	**Error (%)**
Kahane, 2016 [20]	Harmonic detection with CS	-		-		-	-		-	-
Yang, 2016 [21]	Harmonic detection with CS	-		-		-	-		-	1.8
Majidi, 2017 [19]	Distribution system state estimation with CS		-	-	-		-		-	0.50
Palczynska, 2020 [17]	Harmonic detection with CS		-	-	-	-			-	1.00
Mukherjee, 2020 [26]	Estimation for fault analysis with CS		-	-	-		-		-	-
Daponte, 2021 [22]	A reduced-code method for integral nonlinearity testing in DACs	-		-	-		-			0.044 RMSE
Andras, 2021 [24]	Compressed sensing with continuous parametric reconstruction	-	-	-	-	-	-			-
Niu, 2022 [27]	Harmonic detection with CS	-	-			-	-		-	0.15
Present work	Harmonic detection with CS	-	-		-		-			1.78

**Table 3:** Notations used in this article.

Dimensions	
*K*	Number of nonzero entries in a K-sparse vector s
*m*	Number of data snapshots (i.e., columns of X)
*n*	Dimension of the state, x ε Rn
*p*	Dimension of the measurement or output variable, y ε Rp
Vectors	
*s*	Sparse vector, s ε Rn
*x*	Original signal
$x^{,}$	Reconstructed signal
*y*	Vector of measurements, y ε Rp
Matrix	
*C*	Measurement matrix
Θ	Dictionary matrix
Ψ	Orthonormal basis (e.g., Fourier, wavelet, Gabor, etc.)
$\Psi^{\prime }$	Inverse orthonormal basis (e.g., Fourier, wavelet, Gabor, etc.)
Φ	Projection matrix
Norms	
${∥.∥}_{0}$	L0 pseudo-norm of a vector x, the number of nonzero elements in x
${∥.∥}_{1}$	L1-norm of a vector x given by ${∥x∥}_{1}=\sum_{i=1}^{n}\left(Xi^{2}\right)$
${∥.∥}_{2}$	L2-norm of a vector x given by ${∥x∥}_{2}=\sqrt{\sum_{i=1}^{n}\left(Xi^{2}\right)}$
Transform	
DCT	Discrete cosine transform
DFT	Discrete Fourier transform
DGT	Discrete Gabor transform
DRT	Discrete Radon transform
DWT	Discrete wavelet transform
SVD	Singular-value decomposition
IDCT	Discrete cosine inverse transform
Harmonic	
THD	Total harmonic distortion
$I_{1}$	RMS value of the fundamental component
In	RMS value of the nth harmonic voltage
*h*	Harmonic (2,3,4…)
Error	
$I_{approx}$	Approximation value
*I*	Pattern value
δ	Percent error
η	Relative error
ε	Absolute error

**Table 4:** Parameters of the standard signal.

Parameter	Measure
THD I	74.25%
Fundamental Amplitude	64.65 mA
Harmonic 3	37.65 mA
Harmonic 5	16.40 mA
Harmonic 7	15.94 mA
Harmonic 9	10.43 mA
Harmonic 11	8.49 mA
Harmonic 13	7.87 mA
Harmonic 15	7.15 mA
Harmonic 17	6.71 mA
Harmonic 19	3.94 mA
Harmonic 21	3.04 mA
Number of samples per cycle	64

## Data Availability

Not applicable.
